# Achieving More Optimal Lipid Control with Non-Statin Lipid Lowering Therapy

**DOI:** 10.1007/s11883-025-01280-4

**Published:** 2025-02-15

**Authors:** Stephen J. Nicholls, Adam J. Nelson

**Affiliations:** Victorian Heart Institute, Monash University, 631 Blackburn Rd, Clayton, Melbourne, Australia

**Keywords:** Lipid lowering, Cardiovascular risk, PCSK9, Ezetimibe, Bempedoic acid

## Abstract

**Purpose of Review:**

The use of statins has transformed approaches to the prevention of cardiovascular disease. However, many patients remain at a major risk of experiencing cardiovascular events, due to a range of factors including suboptimal control of low-density lipoprotein cholesterol (LDL-C). Accordingly, there is an ongoing need to develop additional strategies, beyond the use of statins, to achieve more effective reductions in cardiovascular risk.

**Recent Findings:**

Genomic studies have implicated the causal role of LDL in atherosclerosis and identified that polymorphisms influencing factors involved in lipid metabolism influence both the level of LDL-C and cardiovascular risk. These findings have highlighted the potential for cardiovascular benefit from development of therapies targeting these factors and incremental benefit when used in combination with statins. Clinical trials have demonstrated that these new agents have favourable effects on both atherosclerotic plaque and cardiovascular events. Additional work has sought to improve intensification of statin therapy and adherence with lipid lowering therapy, to achieve more effective cardiovascular prevention via lipid lowering.

**Summary:**

Emerging therapies, beyond statins, have the potential to optimise lipid levels and play an effective role in the prevention of cardiovascular disease.

## Introduction

Low-density lipoproteins play a pivotal role in the pathogenesis of atherosclerotic cardiovascular disease. This has been well established by preclinical investigations that demonstrate an association between increasing levels of LDL cholesterol (LDL-C) and atherogenesis, genetic studies that implicate polymorphisms and mutations associated with higher LDL-C levels and cardiovascular risk and by cohort studies that demonstrate a curvilinear relationship between LDL-C and the prospective risk of cardiovascular events [1, 2]. The finding that lowering levels of LDL-C results in a reduction in cardiovascular risk, further supports the importance of LDL-C lowering as a cornerstone for the prevention of cardiovascular disease [3–5]. 2024 represents the thirty-year anniversary of the report of the first large cardiovascular outcomes trial demonstrating clinical benefit with statins in patients with a prior myocardial infarction [6]. While efforts have concentrated on increasing use of statins in clinical practice, more recent advances in lipid therapeutics have focused on the development of additional agents with the potential to both lower LDL-C and cardiovascular risk.

### Statins and Cardiovascular Risk Reduction

Statins are pharmacological inhibitors of 3-hydroxy-3-methylglutaryl coenzyme A (HMGCoA) reductase and are widely used for lowering levels of LDL-C. Mendelian randomization studies have established that the presence of genetic polymorphisms resulting in less HMGCoA reductase associate with lower levels of LDL-C and cardiovascular events [7, 8]. Use of statins have produced cardiovascular benefit across the clinical spectrum from high-risk primary prevention through to those who have had a recent acute coronary syndrome [3–5]. The use of more intensive statin doses, achieving lower LDL-C levels, have produced benefit compared with more moderate lipid lowering [3–5]. In parallel, a range of forms of plaque imaging have permitted evaluation of the impact of statin therapy on atherosclerotic disease. Use of carotid intima-medial thickness [9–11], coronary angiography [12, 13], intravascular ultrasound [14–18] and computed tomography coronary angiography [19–21] have demonstrated that statins can both slow progression of disease or promote plaque regression. More advanced techniques including virtual histology [22–25], magnetic resonance [26], high resolution computed tomography [19–21] and optical coherence tomography [27–33] have extended these findings to demonstrate plaque composition changes consistent with stabilization. The findings of these studies and the direct association between LDL-C lowering and clinical benefit have prompted successive updates to clinical guidelines that link the degree of clinical risk with LDL-C target. Additional analyses have demonstrated an independent association between lowering levels of high sensitivity C-reactive protein (hsCRP) and both slowing of plaque progression [34] and reducing cardiovascular event rates [35] observed with statins. This would support the concept that statins may possess additional properties, beyond their ability to lower LDL-C that may influence cardiovascular risk.

However, several challenges remain regarding the use of statins in clinical practice. Clinical registries demonstrate that patients at high cardiovascular risk are undertreated, both from the perspective of failure to either intensify statin doses or use combination therapy [36–38]. Many patients stop taking their statin within the first year of initiation [39–41], some of which is due to the experience of muscle symptoms or concerns regarding the reported increased risk of developing diabetes [42]. The combination of intensity and adherence to lipid lowering therapy is critical in determining achieved LDL-C levels and potential reductions in cardiovascular risk [38]. When statins are taken in clinical trials, there remains a residual risk of plaque progression [43] and clinical events [44], suggesting that additional approaches are required, in order to achieve more effective prevention of cardiovascular disease.

### Ezetimibe

Ezetimibe inhibits intestinal cholesterol absorption, via its ability to inhibit Niemann-Pick C1-Like 1 (NPC1L1), resulting in the ability to lower LDL-C by approximately 20% [45]. While ezetimibe monotherapy does not lower hsCRP levels, when used in combination with statins the degree of hsCRP lowering is greater than that observed with statin monotherapy [45]. Mendelian randomization studies demonstrate not only less cardiovascular risk in the setting of less NPC1L1, but greater protection in the combination with less HMGCoA reductase, suggesting the potential for incremental clinical benefit using the combination of statins and ezetimibe [7]. 

Clinical trials have demonstrated the impact of therapeutic regimens including ezetimibe on cardiovascular outcomes. The Plaque Regression with Cholesterol Absorption Inhibitor or Synthesis Inhibitor Evaluated by Intravascular Ultrasound (PRECISE-IVUS) trial demonstrated that addition of ezetimibe to atorvastatin produced greater regression of coronary atherosclerosis on serial intravascular ultrasound imaging, compared with atorvastatin monotherapy [46]. The Study of Heart and Renal Protection (SHARP) demonstrated that the combination of simvastatin and ezetimibe reduced the risk of the combination of myocardial infarction, coronary death, non-hemorrhagic stroke or arterial revascularization by 17% compared with placebo in patients with chronic kidney disease, who have yet to experience a cardiovascular event [47]. The Improved Reduction of Outcomes: Vytorin Efficacy International Trial (IMPROVE-IT) demonstrated that the combination of simvastatin and ezetimibe produced a modest (6.4%), but significant, reduction in the combination of cardiovascular death, myocardial infarction, unstable angina requiring hospitalization, coronary revascularization or stroke, compared with simvastatin monotherapy in patients with an acute coronary syndrome [48]. The clinical benefit of these studies directly associated with the extent of LDL-C lowering and confirmed the use of ezetimibe as an important approach to achieving more effective lipid control in high cardiovascular risk patients.

### PCSK9 Inhibitors

Proprotein convertase subtilisin kexin type 9 (PCSK9) plays a role in LDL metabolism. Synthesized within the liver, limits removal of LDL particles from the circulation, via binding to the LDL receptor and preventing its recirculation to the hepatocyte surface [49]. Early genetic studies identified gain of function PCSK9 mutations as a monogenic cause of familial hypercholesterolemia, while loss of function mutations was demonstrated in healthy individuals with very low LDL-C levels [49]. Mendelian randomization studies similarly revealed cardiovascular protection with low PCSK9 levels, which was greater in the presence of less HMGCoA reductase [50]. Early clinical studies of PCSK9 inhibitory monoclonal antibodies demonstrated dose-dependent lowering of LDL-C by up to 60%, when used as monotherapy or in combination with statins [51, 52]. Tolerability was good, with mild injection site reactions experienced by some patients. This presented the opportunity to both enable more patients to achieve their LDL-C goals and for high-risk patients to achieve very low LDL-C levels. These benefits extended to the setting of familial hypercholesterolemia [53, 54], particularly the most severe forms in which the requirement for LDL apheresis was decreased [53, 55].

Serial plaque imaging studies made several seminal discoveries regarding the impact of adding a PCSK9 inhibitor to statin therapy [56]. The Global Assessment of Plaque Regression with a PCSK9 Antibody as Measured by Intravascular Ultrasound (GLAGOV) trial demonstrated that addition of the PCSK9 inhibitor, evolocumab, to existing statin therapy for 18 months produced plaque regression compared with continuing statin monotherapy [57]. Greater degrees of plaque regression were observed in patients achieving the lowest LDL-C levels [57]. The High-Resolution Assessment of Coronary Plaques in a Global Evolocumab Randomized Study (HUYGENS) studied the impact of adding evolocumab to high intensity statin therapy for 12 months on coronary atheroma using multimodality imaging, in patients following an acute coronary syndrome [58]. Serial intravascular ultrasound imaging also demonstrated greater plaque regression with the combination of evolocumab and statin, compared with statin monotherapy [58]. Greater regression was observed in HUYGENS, compared with GLAGOV, despite a shorter treatment period, suggesting potential greater modifiability of atherosclerosis in patients with an acute coronary syndrome. Optical coherence tomography imaging demonstrated that the combination of statin and PCSK9 inhibitor produced greater thickening of the fibrous cap and reduction in plaque lipid and macrophages, consistent with plaque stabilization. Similar findings were observed in the Effects of the PCSK9 Antibody Alirocumab on Coronary Atherosclerosis in Patients with Acute Myocardial Infarction (PACMAN-AMI) study, in which addition of alirocumab to a statin produced plaque regression on intravascular ultrasound, reductions in plaque macrophages on optical coherence tomography and lipid cores on near infrared spectroscopy [59]. While these trials involved at least 12 months of treatment, observational studies have suggested that plaque stabilization features begin to appear as early as 4 weeks after commencement of combination therapy with a PCSK9 inhibitor and a statin (10.1016/j.jjcc.2019.08.002). The Effect of Evolocumab on Coronary Plaque Characteristics in Stable Coronary Artery Disease: a Multimodality Imaging Study (YELLOW 3) extended the findings of plaque stabilization with statin and evolocumab to patients with stable disease [60]. 

The impact of PCSK9 inhibitory monoclonal antibodies on cardiovascular outcomes have been investigated in three clinical trials. The Further Cardiovascular Outcomes Research with PCSK9 Inhibition Subjects with Elevated Risk (FOURIER) study demonstrated that addition of evolocumab to statin therapy produced a 15% reduction in the risk of the composite of cardiovascular death, myocardial infarction, stroke, hospitalization for unstable angina or coronary revascularization in patients with stable atherosclerotic cardiovascular disease [61]. A direct association was observed between achieved LDL-C levels and the rate of cardiovascular events [61]. No increase in adverse events was observed in those patients achieving the lowest LDL-C levels [62]. In addition, a cognitive function substudy demonstrated no adverse impact of treatment with evolocumab [63]. *Post hoc* analyses demonstrated a greater absolute risk reduction with evolocumab in patients with multivessel disease, polyvascular disease, recurrent events and multiple risk factors [8, 64, 65]. The long-term extension study demonstrated increasing benefit, with evidence of a significant reduction in cardiovascular death [66]. An ongoing clinical trial is evaluating the impact of evolocumab on cardiovascular risk in patients deemed to be at high risk, yet have not experienced a clinical event (10.1016/j.ahj.2023.12.004).

The Evaluation of Cardiovascular Outcomes After an Acute Coronary Synrdome During Treatment with Alirocumab (ODYSSEY Outcomes) study demonstrated that addition of alirocumab to statin therapy produced a 15% reduction in the risk of the composite of coronary heart disease death, myocardial infarction, ischemic stroke or unstable angina requiring hospitalization in patients with an acute coronary syndrome within the preceding 4 to 52 weeks [67]. A nominal reduction in mortality was observed [67]. The study design required that patients achieving very low LDL-C levels (< 0.39 mmol/L or 15 mg/dL) underwent blinded back titration of therapy or swapping to placebo. These patients demonstrated lower rates of cardiovascular events, suggesting that even short periods of achieving very low LDL-C levels could produce a legacy effect on cardiovascular risk [68]. Subsequent analyses of the ODYSSEY study demonstrated that lowering levels of Lp(a) with alirocumab, independently associated with its clinical benefit [69]. 

Monoclonal antibodies bind circulating PCSK9 that has been synthesized in the liver. An alternative approach involves inhibition of hepatic PCSK9 synthesis, leading to the development of short interfering RNA interference (siRNA) therapeutics that are selectively targeted to the hepatocyte [70–72]. Inclisiran is the first siRNA targeting PCSK9 to advance in clinical development. Early studies have demonstrated that inclisiran reduces LDL-C levels by more than 45%, but also with good durability of effect. LDL-C lowering by more than 38% has been observed up to 6 months following a single injection of inclisiran, providing the opportunity to develop twice yearly administration [70–73]. This has important implications for the treatment of patients, where adherence may be a challenge. Longer term administration has demonstrated both sustained efficacy and good tolerability by patients [74]. The impact of inclisiran on cardiovascular events is currently being evaluated in three ongoing clinical trials, one in the primary prevention setting and two in patients with clinically manifest atherosclerotic cardiovascular disease.

Most jurisdictions have limited access to these agents, due to their cost, which has influenced cost effectiveness analyses. This typically involves delaying administration to the outpatient setting, downstream of a clinical event. Clinical trials have investigated the impact of early initiation of either evolocumab [75] or inclisiran [76] in the setting of an acute coronary syndrome. These studies have demonstrated good tolerability and early lipid lowering, which then subsequently translates to favorable effects on coronary atheroma with more sustained use. The impact of early administration of evolocumab in the acute coronary syndrome patient on cardiovascular outcomes is being evaluated in an ongoing clinical trial.

Ongoing work is aiming to develop alternative PCSK9 inhibitor formulations for administration. Lerodalcibep is a recombinant fusion protein of adnectin, a PCSK9 gene binding domain, and human serum albumin. Administered in small volumes monthly, lerodalcibep reduced LDL-C levels by up to 65% in patients with heterozygous familial hypercholesterolemia [77]. PCSK9 inhibitors have also been developed in an oral form, with a macrocyclic peptide lowering LDL-C in a dose-dependent fashion by up to 61% [78]. These agents will need to be evaluated in larger and longer trials.

### Bempedoic Acid

Bempedoic acid is an oral lipid lowering agent that inhibits ATP citrate lyase, a factor involved in hepatic cholesterol synthesis [79]. Mendelian randomization demonstrated that polymorphisms associated with less ATP citrate lyase resulted in lower cardiovascular risk, which was additive to lower levels of HMGCoA reductase [80]. Like statins, bempedoic acid lowers LDL-C levels by promoting upregulation of hepatic LDL receptors and removal of LDL from the circulation. Bempedoic acid is ingested in an inactive form, with the isozyme responsible for activation present in the liver, but not skeletal muscle [79, 81, 82]. In theory, bempedoic acid should produce LDL-C lowering, without the myalgia symptoms experienced by up to 20% of patients treated with a statin. Early clinical studies demonstrated that bempedoic acid lowered LDL-C by 15–25% and hsCRP by 20–30%, in studies conducted in both statin intolerant patients and those receiving maximally tolerated, with lesser effects observed in those taking a statin [83–90]. Further studies had demonstrated LDL-C lowering by 38–45% with the combination of bempedoic acid and ezetimibe [91] and by 64% with the combination of low dose statin, bempedoic acid and ezetimibe [92]. The CLEAR Outcomes study was conducted in patients at high risk of a cardiovascular event, with elevated LDL-C levels and documented statin intolerance. Treatment with bempedoic acid produced a 13% reduction in the risk of cardiovascular death, myocardial infarction, stroke or coronary revascularization [93–96]. A greater benefit was observed in patients who had yet to experience a clinical event [97]. Bempedoic acid treated patients demonstrated a greater likelihood of gout and liver enzyme abnormalities [93, 94]. 

### CETP Inhibitors

Cholesteryl ester transfer protein (CETP) plays an important role in lipid metabolism, facilitating the exchange of esterified cholesterol from high-density lipoprotein (HDL) to LDL and very low-density lipoprotein (VLDL) particles [98–100]. Early development of CETP inhibitors was stimulated on the basis of their ability to raise HDL cholesterol (HDL-C), yet several clinical trials proved disappointing with either toxicity [101] or no impact on cardiovascular outcomes [102, 103]. While one CETP inhibitor, anacetrapib, did reduce cardiovascular risk, the benefit was modest and not related to HDL-C raising, rather due to lowering of non-HDL-C [104, 105]. This complemented studies from Mendelian randomization, which demonstrated that genetic polymorphisms associated with less CETP also resulted in less cardiovascular events, directly related to lower levels of LDL-C [106–109]. Additional analyses demonstrated additive effects of having less CETP and HMGCoA reductase. This led to a rethink on development of CETP inhibitors, focusing on their ability to lower LDL-C levels, rather than raising HDL-C. Studies of the potent CETP inhibitor obicetrapib have demonstrated LDL-C lowering by 40–50% as monotherapy and in combination with high-intensity statin therapy, in addition to lowering triglyceride by up to 11% and Lp(a) by up to 56% and raising HDL-C levels by up to 165% [110–112]. The impact of obicetrapib on cardiovascular outcomes is currently being evaluated in a large clinical trial of high cardiovascular risk patients with elevated LDL-C levels [113]. 

### Additional Approaches to Lowering LDL-C

A number of additional therapeutic approaches are used in clinical practice for lowering LDL-C in patients with severe forms of familial hypercholesterolemia. Mipomersen is an antisense oligonucleotide targeting apolipoprotein B-100, with LDL-C lowering by up to 36% [114, 115]. Lomitapide inhibits microsomal transfer protein, essential for assembly of VLDLs in the liver and chylomicrons in the intestine, with LDL-C lowering by 50% [116]. Both of these agents result in increases in hepatic fat content and liver enzymes [114–116]. Angiopoietin-like 3 (ANGPTL3) influences lipid metabolism in a number of ways. As an inhibitor of lipoprotein lipase, early studies of ANGPTL3 inhibition primarily focused on their potential to influence triglyceride levels [117]. However, ANGPTL3 inhibition also has the potential to lower LDL-C levels, via a mechanism independent of LDL receptor expression [117]. Genetic studies have demonstrated that loss of function ANGPTL3 variants associate with lower levels of triglycerides, LDL-C and cardiovascular risk [118]. Administration of the ANGPTL3 inhibitory monoclonal antibody, evanicumab, was well tolerated and produced LDL-C lowering by 49% [119]. Novel ANGPTL3 inhibitors aimed at targeting RNA are in clinical development, primarily on the basis of their ability to lower triglyceride levels [120, 121]. They may provide an additional approach to treating LDL-C in the resistant patient. Apheresis remains a therapeutic option for treatment of severe familial hypercholesterolemia, which is refractory to treatment with conventional therapies. While effective, it is a costly and invasive therapeutic option, stimulating the search to develop alternative approaches for patients [122]. 

### Models of Care

Prescribing more intensive lipid lowering regimens can only be effective in cardiovascular prevention if patients remain adherent with therapy. The combination of greater adherence to more intensive lipid lowering therapy results in more effective lowering of both LDL-C and cardiovascular risk [38]. Accordingly, approaches to facilitate shared decision making by both patient and clinician, with a view to improving long term therapeutic adherence is critical to more effectively prevent the risk of cardiovascular events [123]. Educational tools that assist in demonstrating the level of risk for patients can assist in decision making, whether they distil risk scores or use additional information from imaging or genetic scores. Additional digital tools, such as text messages or use of chatbots, can assist in promoting long term adherence to therapies. Efforts are underway to reinstate LDL-C measurements as a quality metric for patients with acute coronary syndromes, as an approach to ensuring more effective lipid management [124]. An alternative approach to achieving more effective long term lipid control involves the use of combination therapy, with lower statin doses, to minimise drug discontinuation and maintenance of cardiovascular risk lowering [125]. More work is required in each of these areas to develop effective models of care that can be integrated into local management plans and used in a fit for purpose strategy with patients.

### Gene Editing

Advances in gene editing now permit selective targeting of gene editing of factors involved in lipid metabolism within the hepatocyte. Early human studies of gene editing have targeted both PCSK9 and ANGPTL3, with evidence of durable and robust LDL-C lowering in non-human primates [126]. While longer studies are required to characterize both efficacy and safety of these interventions, they do present the potential for a once in a lifetime approach to management of dyslipidemia.

## Conclusion

While statins have played a major role in lowering levels of LDL-C and cardiovascular events, residual risk and challenges with achieving treatment targets highlight the need to develop additional lipid lowering agents. Genetic studies have identified a number of pharmacological targets that have led to new therapies, which favorably modulate lipid levels in the blood, plaque burden and composition within the artery wall and ultimately cardiovascular risk. The use of these therapies, both in combination with statins and as monotherapy in patients unable to tolerate statins, have the potential to achieve more effective lipid lowering and prevention of cardiovascular disease.

### Key References

1. Raal F, Fourie N, Scott R, et al.: Long-term efficacy and safety of lerodalcibep in heterozygous familial hypercholesterolaemia: the LIBerate-HeFH trial. Eur Heart J 2023, 44(40):4272–4280. New injectable form of PCSK9 inhibitor in development.
2. Ballantyne CM, Banka P, Mendez G, et al.: Phase 2b Randomized Trial of the Oral PCSK9 Inhibitor MK-0616. J Am Coll Cardiol 2023, 81(16):1553–1564. First human experience of an oral PCSK9 inhibitor.
3. Nissen SE, Lincoff AM, Brennan D, et al.: Bempedoic Acid and Cardiovascular Outcomes in Statin-Intolerant Patients. N Engl J Med 2023, 388(15):1353–1364. Impact of bempedoic acid on cardiovascular outcomes in patients with statin intolerance.
4. Nicholls SJ, Ditmarsch M, Kastelein JJ, et al.: Lipid lowering effects of the CETP inhibitor obicetrapib in combination with high-intensity statins: a randomized phase 2 trial. Nat Med 2022, 28(8):1672–1678. Effects of a potent CETP inhibitor on atherogenic lipid levels in patients treated with high-intensity statins.

## Data Availability

No datasets were generated or analysed during the current study.
